# Induced expression of GINS complex is an essential step for reactivation of quiescent stem-like tumor cells within the peri-necrotic niche in human glioblastoma

**DOI:** 10.1007/s00432-018-2797-z

**Published:** 2018-11-21

**Authors:** Tokuhiro Kimura, Dan Cui, Hiroo Kawano, Chihiro Yoshitomi-Sakamoto, Nobuyuki Takakura, Eiji Ikeda

**Affiliations:** 1 Department of Pathology, Yamaguchi University Graduate School of Medicine, 1-1-1 Minami-Kogushi, Ube, Yamaguchi 755-8505 Japan; 2 Department of Basic Laboratory Sciences, Yamaguchi University Graduate School of Medicine, 1-1-1 Minami-Kogushi, Ube, Yamaguchi 755-8505 Japan; 3 Department of Signal Transduction, Research Institute for Microbial Diseases, Osaka University, 3-1 Yamadaoka, Suita, Osaka 565-0871 Japan

**Keywords:** GINS complex, Glioblastoma, Niche, Necrosis, Quiescence, Proliferation

## Abstract

**Purpose:**

Glioblastoma is still intractable despite the progress in therapies, and the intractability is attributable to a minor population of stem-like tumor cells. As a niche harboring quiescent stem-like tumor cells with potentially high tumorigenicity, we have specified an area around large ischemic necrosis, termed ‘peri-necrotic niche’, in glioblastoma. In this study, the behavior of tumor cells inside and outside the peri-necrotic niche was analyzed to find out molecules responsible for reactivation of quiescent stem-like tumor cells to proliferate outside the niche.

**Methods:**

Expression of Ki-67 and GINS complex composed of SLD5, PSF1, PSF2 and PSF3 was analyzed by immunohistochemistry in human glioblastoma tissue samples. Proliferation assays, immunoblotting and siRNA experiments were performed using a glioblastoma cell line.

**Results:**

Immunohistochemical analysis revealed quiescent and proliferative phenotypes of tumor cells inside and outside the niche, respectively, and the proliferation was spatially correlated with the expression of GINS components in tumor cells. To mimic the tissue microenvironment inside versus outside the niche, glioblastoma cells were cultured under hypoxic versus normoxic conditions, or without versus with serum. Quiescence and proliferation of tumor cells were reversibly determined by the microenvironment inside and outside the niche, respectively, and proliferative activities paralleled the expression levels of GINS components. Furthermore, the reactivation of proliferation after reoxygenation or serum replenishment was suppressed in quiescent tumor cells with PSF1 knockdown.

**Conclusions:**

These findings indicate the essential role of GINS complex in the switch between quiescence and proliferation of tumor cells inside and outside the peri-necrotic niche.

## Introduction

Prognosis of patients with glioblastoma is unfavorable regardless of many studies which analyzed the genetic abnormalities as well as the pathophysiology of glioblastoma (Ohgaki et al. 2004). Accumulative genetic analyses have figured out a couple of genetic profiles which are closely correlated with the biological behaviors including the sensitivity to treatments, and, therefore, the clinicians tend to make account of genetic information rather than histopathological diagnosis of gliomas (Brat et al. 2015; Eckel-Passow et al. 2015). However, genetic studies have not been successful in offering the targets to establish the therapeutic strategies for eradication of tumor cells. Other approaches to overcome the poor prognosis of glioblastoma patients have been focused on a subpopulation of tumor cells responsible for its intractability. It is generally accepted that unfavorable prognosis of glioblastoma is attributable to a minor population of tumor cells with stem cell-like properties which would be, therefore, the candidates for therapeutic targets (Chen et al. 2012b; Sundar et al. 2014). Putative tumor stem cells are shown to reside in the certain microenvironment within a tumor which is called ‘niche’ (Chen et al. 2012b; Sundar et al. 2014). Recently, we have specified an area around the large ischemic necrosis as a niche harboring quiescent stem-like tumor cells with potentially high tumorigenicity (Ishii et al. 2016). Quiescent stem-like tumor cells are concentrated in the zone between ischemic necroses and blood vessels and are preferentially closer to the necroses rather than to the blood vessels, which we termed ‘peri-necrotic niche’. Our in vitro data with a glioblastoma cell line demonstrated that stemness as well as quiescence are induced and kept in tumor cells while they stay in the peri-necrotic niche, and those properties are not intrinsic natures of a certain population of tumor cells. For growth of tumors including recurrence after chemotherapy, stem-like tumor cells are required to acquire the proliferative activity in the microenvironment, outside of the peri-necrotic niche, with increased oxygen concentration, plenty of nutrients and so forth. Therefore, the molecules responsible for activating the stem-like tumor cells when they go outside the peri-necrotic niche could be the targets for new therapies to overcome the poor prognosis of patients by keeping tumors in dormant state, even if there remain tumor cells alive after therapies.

GINS complex, an abbreviation of ‘go-ichi-ni-san’ which corresponds to Japanese numbers 5-1-2-3, is a protein complex comprised of SLD5, PSF1, PSF2 and PSF3 and works as a DNA helicase with CDC45 as well as MCM2-7 (CMG complex) to replicate DNA (Aparicio et al. 2009; MacNeill 2010). Several lines of evidence have revealed the involvement of GINS complex in the reconstruction of bone marrow (Ueno et al. 2009) as well as the tumorigenicity of neoplasms such as lung and colon carcinoma and so forth (Nagahama et al. 2010a, b; Nakahara et al. 2010; Zhang et al. 2015), suggesting the critical roles of GINS in regulating the proliferative behavior of cells with stemness. These findings have prompted us to hypothesize that GINS complex is the switch for reactivating quiescent stem-like tumor cells to leave the peri-necrotic niche as well as proliferate and consequently reconstitute the glioma masses. Here, we demonstrate the data showing that, in response to the changes in tissue oxygen concentration and amount of nutrients, GINS plays the critical roles in phenotypic changes of glioblastoma cells inside and outside of peri-necrotic niche which are quiescent and proliferative, respectively.

## Materials and methods

### Clinical samples

We analyzed 8 cases of glioblastoma [median (range) of age, 67 (51–81) years; male:female ratio, 4:4] which had been operated at Yamaguchi University Hospital. In all the cases, histological diagnosis was reviewed and confirmed as glioblastoma, NOS according to the latest WHO Classification (Louis et al. 2016). Formalin-fixed paraffin-embedded tissue sections (4-µm thick) were prepared for immunohistochemical analyses. This study was approved by the Institutional Review Board, Yamaguchi University Hospital, and performed in accordance with the 1964 Helsinki declaration and its later amendments or comparable ethical standards. Informed consent was obtained from all individual participants included in the study.

### Immunohistochemistry

Deparaffinized sections were pretreated for antigen retrieval by boiling in the solutions: antigen retrieval solution, pH 9 (Nichirei Biosciences, Tokyo, Japan) for Ki-67, PSF1, PSF3, HIF-1α and NANOG; 10 mM citrate buffer, pH 6 for PSF2, SLD5 and MCM2; Immunosaver solution (Nissin EM, Tokyo, Japan) for CDC45. Endogenous peroxidase activity was blocked with peroxidase-blocking solution (Dako, Glostrup, Denmark). Sections were incubated overnight at 4 °C with rabbit monoclonal antibody against Ki-67 (1:1000; clone EPR3610; Epitomics, Burlingame, CA, USA), rat monoclonal antibody against PSF1 (1:300; clone aho57.2; GeneStem, Osaka, Japan), rabbit polyclonal antibody against PSF2 (1:3000; HPA057285, Atlas Antibodies, Stockholm, Sweden), mouse monoclonal antibody against PSF3 [1:500; clone aps3.2 (Nagahama et al. 2010a)], rabbit polyclonal antibody against SLD5 (1:1000; ab101346, Abcam, Cambridge, UK), rabbit monoclonal antibody against MCM2 (1:1000; clone D7G11, Cell Signaling Technology, Danvers, MA, USA) and rabbit monoclonal antibody against CDC45 (1:1000; clone EPR5759, Abcam), mouse monoclonal antibody against HIF-1α (1:20; clone 54/HIF-1α; BD Biosciences, Franklin Lakes, NJ, USA) and goat polyclonal antibody against NANOG (1:250; Novus Biologicals, Littleton, CO, USA). After the subsequent reaction with horseradish peroxidase (HRP)-conjugated secondary antibodies (EnVision+, Dako, for mouse and rabbit primary antibodies; Simple stain, anti-rat, Nichirei Biosciences, for rat primary antibodies; ImmPRESS, anti-goat Ig, Vector Laboratories, Burlingame, CA, USA, for goat primary antibodies), color was developed with 3,3′-diaminobenzidine (DAB) and sections were counterstained with hematoxylin. For negative control experiments, primary antibody was replaced by non-immune immunoglobulin (Dako).

### Quantitative analyses of immunostaining results

**Fig. 1:**
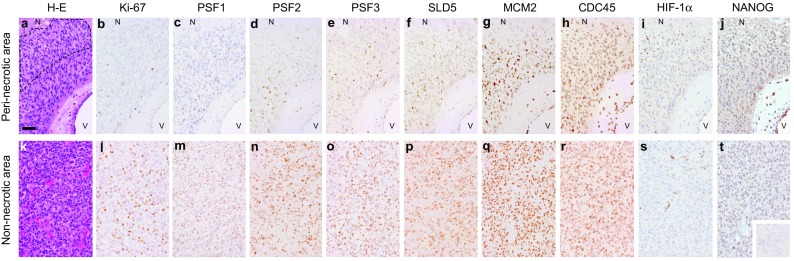
Immunohistochemistry for Ki-67, GINS complex components (PSF1, PSF2, PSF3 and SLD5), MCM2, CDC45, HIF-1α and NANOG in human glioblastoma tissues. Distribution of immunoreactive cells in the peri-necrotic area (**a**–**j**) and the non-necrotic area (**k**–**t**) is shown. Dashed line **a** indicates the region of peri-necrotic niche, the zone which is between large ischemic necrosis (*N*) and nearest blood vessels (*V*) and is closer to the necrotic tissues than to the blood vessels. Hematoxylin-eosin stain (**a, k**), immunostain for Ki-67 (**b, l**), PSF1 (**c, m**), PSF2 (**d, n**), PSF3 (**e, o**), SLD5 (**f, p**), MCM2 (**g, q**), CDC45 (**h, r**), HIF-1α (**i, s**), NANOG (**j, t**) and negative control stain using non-immune immunoglobulins (**t**, inset). *N* large ischemic necrosis, *V* blood vessel. Scale bar for **a**–**t**, 50 µm (**a**)

Regions of peri-necrotic niche were defined as the zones which are located between large ischemic necroses and nearest blood vessels and were closer to the necrotic tissues than to the blood vessels (Ishii et al. 2016) (Fig. 1a). Non-necrotic areas were defined as viable tumor areas at a distance of 2 mm from large ischemic necroses (Fig. 1k). In each case, representative 3 microscopic fields of the peri-necrotic niche and non-necrotic area were selected, and immunoreactivity for antibodies was evaluated for a total of at least 300 cells. Labeling index, a percentage of immunoreactive cells in total cells, was recorded and compared in the peri-necrotic niche and non-necrotic area in each case.

### Cell culture under hypoxic or serum-free conditions

The human glioblastoma cell line T98G was obtained from American Type Culture Collection (Manassas, VA, USA). Cells were grown in minimum essential medium (MEM; Gibco, Life Technologies, Carlsbad, CA, USA) supplemented with 10% (v/v) fetal bovine serum (FBS; HyClone, South Logan, UT, USA). Cells (4 × 10^5^/dish) were seeded in 3.5-cm-diameter culture dishes 24 h before the exposure to hypoxic or serum-free conditions as well as the transfection of small interfering RNAs (siRNAs). The cells were treated by hypoxia or serum deprivation which is thought to constitute the microenvironment of peri-necrotic niche. A multi-gas incubator with an O_2_ control system (APM-50DR, ASTEC, Fukuoka, Japan) was used to generate the hypoxic culture conditions. For hypoxic treatment, the cells were incubated in MEM with 10% FBS for 24 or 72 h under hypoxia (5% CO_2_ and 1% O_2_ balanced with N_2_). For the experiments of reoxygenation, the cells treated with hypoxia for 72 h were further cultured for 24 h under normoxia (5% CO_2_ and 95% atmospheric air). For serum-free treatment, the cells were incubated in serum-free MEM for 24 or 72 h. To release the cells from serum-free condition for 72 h, they were further cultured for additional 24 h in MEM containing 10% FBS.

### Immunoblotting

Cells were washed twice with ice-cold PBS and lysed in 25 mM Tris–HCl, pH 7.4, 150 mM NaCl, 1% Triton X-100, 0.1% SDS and protease inhibitor cocktail (Thermo Fisher Scientific, Waltham, MA, USA). The total cell lysates (40 µg protein/lane) were resolved by SDS–polyacrylamide gel electrophoresis under reducing conditions. The proteins were transferred onto polyvinylidene difluoride membranes and subjected to immunoblotting using primary antibodies as follows: anti-PSF1 (A304-170A-M, Bethyl Laboratories, Montgomery, TX, USA), anti-PSF2 (HPA057285), anti-PSF3 (aps3.2), anti-SLD5 (ab101346) and anti-MCM2 (D7G11) antibodies. Anti-β-Tubulin (clone 9F3, Cell Signaling Technology) antibody was used as a loading control. HRP-conjugated secondary antibodies (EnVision+) and Pierce ECL Western Blotting Substrate (Thermo Fisher Scientific) were used.

### Proliferation assay

Proliferation of cultured cells was evaluated by Ki-67 immunostain and bromodeoxyuridine (BrdU) incorporation. Ki-67 immunostaining labels cells in G_1_, S, G_2_ and M phases of the cell cycle, while cells in G_0_ phase (for example, quiescent cells) are negative for Ki-67. As for BrdU incorporation, only cells in S phase are labeled.

For Ki-67 immunostaining, cells in 3.5-cm-diameter culture dishes were washed with PBS, fixed with 10% neutral buffered formalin and permeabilized with 0.1% Triton X-100 for 10 min. For BrdU incorporation assay, cells were cultured with BrdU (10 µM; Sigma–Aldrich, St. Louis, MO, USA) for 30 min, and then washed with PBS, fixed with 10% neutral buffered formalin, permeabilized with 0.1% Triton X-100 and treated with 2N HCl at 37 °C for 30 min. After overnight incubation at 4 °C with mouse anti-Ki-67 antibody (1:100; clone MIB-1, Dako) or mouse anti-BrdU antibody (1:300; clone Bu20a, Dako), HRP-conjugated secondary antibody (ImmPRESS, anti-mouse Ig, Vector Laboratories) was applied and color was developed with DAB. Cells were counterstained with hematoxylin. A percentage of immunostained cells in total cells was calculated as Ki-67 or BrdU labeling index.

### Double immunofluorescence staining of cultured cells

Double immunofluorescence staining for HIF-1α and NANOG was performed. Cells in 3.5-cm-diameter culture dishes were washed with PBS, fixed with 10% neutral buffered formalin and permeabilized with 0.1% Triton X-100 for 10 min. Then, they were reacted with mouse monoclonal antibody against HIF-1α (1:20; clone 54/HIF-1α; BD Biosciences) and goat polyclonal antibody against NANOG (1:250; Novus Biologicals) overnight at 4 °C. After washing with PBS, they were incubated with Alexa Fluor 546 donkey anti-mouse IgG and Alexa Fluor 488 donkey anti-goat IgG (1/200 dilution each; Thermo Fisher Scientific) for double staining for HIF-1α and NANOG at room temperature under light protection for 1 h. For the staining of nuclei, 4′,6-diamidino-2-phenylindole (DAPI) was used. Stained cells were observed under a confocal microscope, LSM510META (Carl Zeiss Jena, Germany).

### Transfection of small interfering RNA

A mixture of 4 siRNAs targeting PSF1 (M-014001-00-0005, siGENOME, SMARTpool) and a mixture of 4 non-targeting siRNAs (D-001206-14-05) were purchased from Dharmacon, Lafayette, CO, USA. Transfection of siRNAs was performed using Lipofectamine RNAiMAX (Thermo Fisher Scientific) according to the manufacturer’s instructions. Hypoxic or serum-free treatment was started 24 h after the transfection.

### Statistical analysis

Paired *t* test was used to compare the labeling indices in the peri-necrotic niches and non-necrotic areas in clinical samples. For the data from experiments using T98G cells, statistical significance was determined by Student’s or Welch’s *t* test. *P* < 0.05 was considered significant.

## Results

### Distribution of GINS components-expressing glioma cells in the peri-necrotic niche

**Fig. 2:**
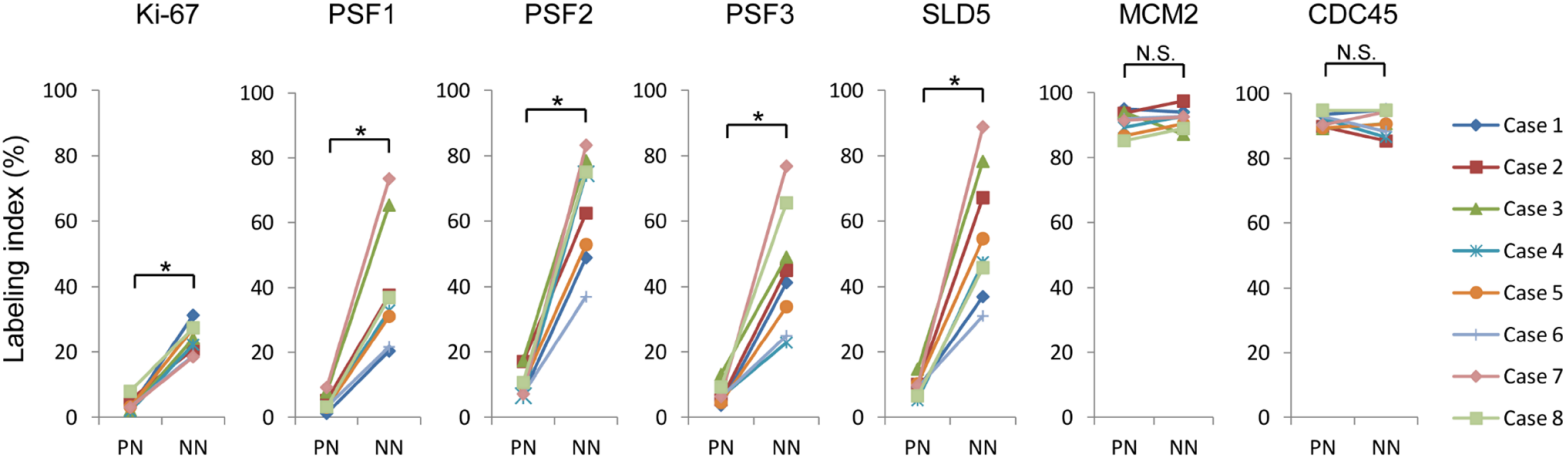
Comparison of the labeling indices for Ki-67, GINS complex components, MCM2 and CDC45 in the peri-necrotic niches (PN) and non-necrotic areas (NN) in glioblastoma cases (*n* = 8). *P < 0.01; *NS* not significant

To surmise the role of GINS complex in the reactivation of quiescent stem-like glioma cells lying dormant in the peri-necrotic niche, the expression of GINS components, PSF1, PSF2, PSF3 and SLD5, in human glioblastoma tissues was analyzed by immunohistochemistry. Distribution of glioma cells expressing MCM2 and CDC45 which confer the helicase activity on GINS complex by forming a CMG complex was also examined. PSF1, PSF2, PSF3 and SLD5 were expressed coordinately with one another in glioma tissues. They were almost undetectable in cells inside the peri-necrotic niche, while their expression was observed in association with Ki-67-positive proliferating glioma cells adjacent to the peri-necrotic niche (Fig. 1a–f) as well as the non-necrotic areas distant from the large ischemic necroses (Fig. 1k–p). In contrast, MCM2 and CDC45 were almost ubiquitously expressed, both inside and outside the peri-necrotic areas, in proliferating as well as non-proliferating glioma cells (Fig. 1g, h, q, r). Glioma cells with the enhanced nuclear accumulation of HIF-1α, which we have reported as a phenotype of quiescent stem-like glioma cells (Ishii et al. 2016), were predominantly localized in the peri-necrotic niche in which cells were negative for GINS components (Fig. 1i, s). NANOG, a stem cell marker, was expressed in glioma cells in the peri-necrotic area, both Ki-67-positive and negative cells, while its expression was diminished in glioma cells in the non-necrotic area (Fig. 1j, t). Quantitative analysis has verified the significant decrease in the frequency of glioma cells expressing GINS components inside the peri-necrotic niche as compared with that outside of the niche, which is attributable to the decrease in Ki-67-positive proliferating cells inside the niche (Fig. 2). Frequency of MCM2- or CDC45-positive glioma cells was independent of their spatial relation to the ischemic necroses (Fig. 2).

### Tissue hypoxia and nutrient deficiency induce the quiescence in glioma cells

**Fig. 3:**
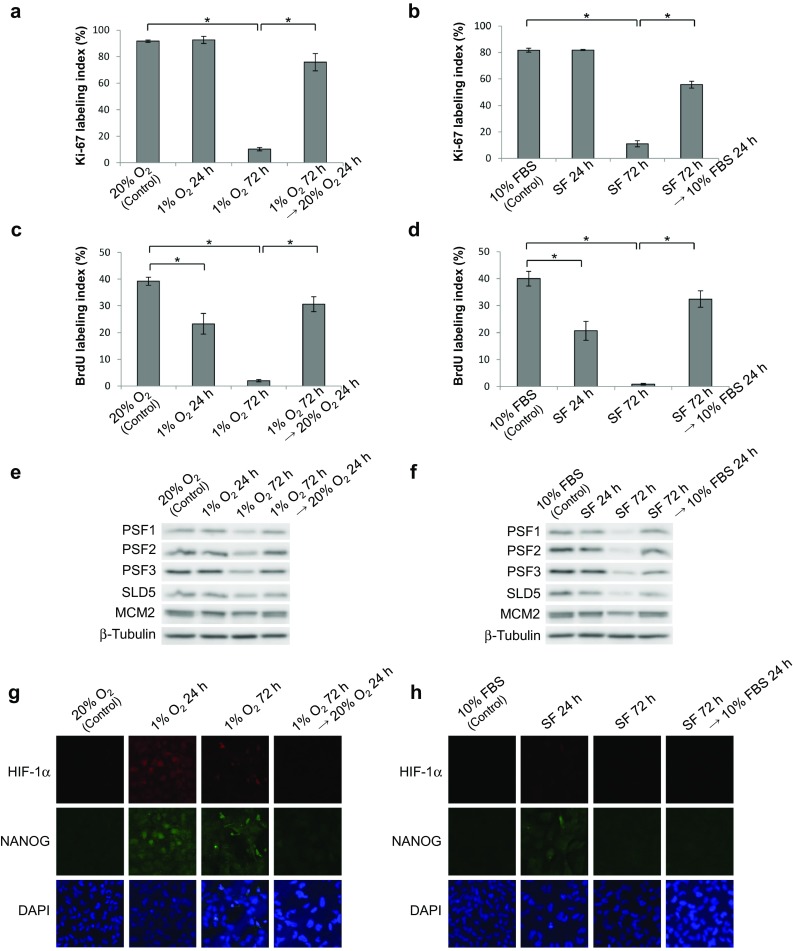
Switching of proliferative/quiescent state by oxygen or serum supply and its correlation with the expression levels of GINS, HIF-1α as well as NANOG in T98G glioblastoma cells. **a**–**d** Changes in Ki-67 (**a, b**) or BrdU (**c, d**) labeling index under hypoxic (**a, c**) or serum-free (**b, d**) treatments. Reoxygenation or serum replenishment after 72-h hypoxia or serum deprivation, respectively, was also performed. Values are expressed as mean ± SD (*n* = 3). *SF* serum-free; *P < 0.01. **e, f** Protein expression of GINS components and MCM2 under changes in oxygen (**e**) or serum (**f**) concentration was examined by immunoblotting. The same hypoxia/reoxygenation and serum deprivation/replenishment treatments as **a** and **b**, respectively, were performed. **g, h** Protein expression and nuclear localization of HIF-1α and NANOG under changes in oxygen (**g**) or serum (**h**) concentration were examined by double immunofluorescence staining

Microenvironment of the peri-necrotic niche is characterized by the decrease in supply of oxygen as well as nutrients, and, therefore, T98G glioma cells were cultured under condition of hypoxia or serum deprivation to reproduce in vitro the peri-necrotic niche of glioma tissues. Quiescence of glioma cells, verified by marked decrease in Ki-67-positive cells, was successfully induced by the hypoxia (1% O_2_) for 72 h or the serum deprivation for 72 h (Fig. 3a, b). Subsequently, the cells in quiescent state under hypoxia or serum deprivation were returned to the condition with normal concentration of oxygen (20%) and serum (10%), respectively. As shown in Fig. 3a, b, the proliferative activity of cells was restored to comparable levels to the levels before exposure to hypoxia or serum deprivation, indicating that the phenotype regarding proliferative activity is determined reversibly by tissue microenvironment including the concentration of oxygen and nutrients. Essentially the same results were also obtained by the experiments in which the proliferative activity was assessed by BrdU incorporation, although the significant decrease in proliferative activity was detectable already 24 h of hypoxia or serum deprivation (Fig. 3c, d).

### Proliferative phenotype of glioma cells is closely correlated with the expression levels of GINS components

Our results of immunohistochemical study on the expression of GINS components in glioma tissues imply that GINS is the switch between quiescent and proliferating phenotypes of glioma cells inside and outside the peri-necrotic niche, respectively. To answer this hypothesis, we reproduced the peri-necrotic niche in vitro with T98G glioblastoma cells and determined the protein expression profiles of GINS components as well as MCM2, one of the associate molecules of GINS, in T98G cells. Interestingly, the protein levels of all the components of GINS (PSF1, PSF2, PSF3 and SLD5) were decreased when quiescence of glioma cells was induced by hypoxia or serum deprivation and restored again by releasing cells from hypoxia and serum deprivation (Fig. 3e, f). By contrast, MCM2 protein levels were almost unchanged although slight decrease was observed in the cells under serum-free condition for 72 h. In addition, the increase in nuclear accumulation of HIF-1α as well as NANOG was observed in the cells cultured under hypoxia for induction of quiescence, while nuclear accumulation of NANOG was detected only slightly in the cells under serum deprivation without HIF-1α accumulation (Fig. 3g, h).

### Induction of GINS expression is essential for the reactivation of quiescent glioma cells in the peri-necrotic niche

**Fig. 4:**
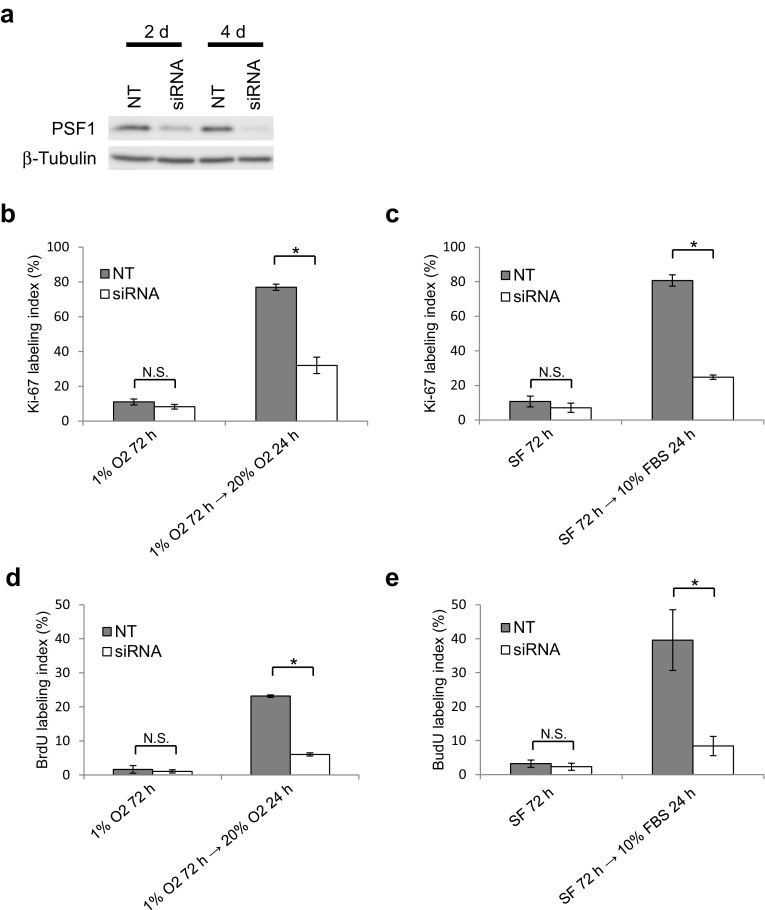
Expression of GINS component PSF1 is essential for the reactivation of quiescent T98G cells by reoxygenation or serum replenishment. **a** Immunoblot showing down-regulation of PSF1 by specific siRNA. Successful knockdown was achieved 2–4 days after siRNA transfection. NT, non-targeting control siRNA. **b**–**e** Response of control (NT) and PSF1-knockdown (siRNA) cells to reoxygenation (**b, d**) or serum replenishment (**c, e**) after 72-h hypoxia or serum deprivation, respectively. Changes in Ki-67 (**b, c**) or BrdU (**d, e**) labeling index were examined. Values are expressed as mean ± SD (*n* = 3). *SF* serum-free; *P < 0.01

We next examined whether the GINS expression induced in glioma cells by the release from the peri-necrotic microenvironment, including hypoxia and nutrient depletion, is essential for the reactivation of cells to proliferate. In T98G cells in which PSF1, as a representative of GINS components, was down-regulated by siRNA transfection (Fig. 4a), the restoration of proliferative activity by reoxygenation after 72 h of hypoxia (Fig. 4b, d) or serum replenishment after 72 h of serum deprivation (Fig. 4c, e) was shown to be suppressed significantly as compared in control cells. These data indicate that the induction of GINS complex is an essential step for reactivation of quiescent stem-like tumor cells in the peri-necrotic niche when they leave the niche and begin to proliferate.

## Discussion

The importance of quiescence of stem-like tumor cells has been increasingly recognized (Chen et al. 2016; Deleyrolle et al. 2011; Dembinski and Krauss 2009; Holyoake et al. 2001; Takeishi et al. 2013). Similar to normal tissue stem cells (Cheung and Rando 2013), subsets of stem-like tumor cells reside within the specific niche in quiescent state, retaining high tumorigenicity responsible for the recurrence and consequently the intractability of glioblastoma (Chen et al. 2012a; Deleyrolle et al. 2011). Recurrence of glioblastoma after chemotherapy was reported to be caused by reactivation of Ki-67-negative quiescent cells (Chen et al. 2012a), and, therefore, the molecules essential for the reactivation of quiescent stem-like tumor cells would be the promising targets for therapeutic strategy to eradicate glioblastoma cells. Recently, we have specified the peri-necrotic area as a niche harboring quiescent stem-like tumor cells with high tumorigenic potential (Ishii et al. 2016). In the present study, immunohistochemistry for Ki-67 has disclosed the highly proliferative phenotype in tumor cells adjacent to and outside the peri-necrotic niche as compared with those inside the niche, suggesting the reversible and dynamic changes in phenotypes of tumor cells occur in response to the microenvironment they are exposed to. In fact, the quiescent T98G cells under hypoxia or serum deprivation which express NANOG were shown to be reactivated reversibly by reoxygenation or serum replenishment. Immunohistochemistry with human glioblastoma tissue samples and the cell biological analyses with T98G cells revealed the close spatial and temporal correlation, respectively, between the expression levels of GINS components and the proliferative phenotypes of tumor cells. Furthermore, the inverse correlation between the expression levels of GINS components and the nuclear accumulation of HIF-1α and NANOG was shown. Therefore, GINS components were considered to be the candidate for the essential molecules which reactivate the quiescent stem-like tumor cells when they leave the peri-necrotic niche and begin to proliferate. It was clearly shown that the quiescent T98G cells with silenced expression of PSF1 could not be reactivated by reoxygenation or serum replenishment, suggesting that the induction of GINS expression is a critical step for the switching of phenotypes of tumor cells from quiescent to proliferative.

Despite their importance in DNA replication, the mechanisms controlling the expression levels of GINS complex are poorly understood both in vitro and in vivo. Although a previous report briefly mentioned their up-regulation after serum stimulation (Hayashi et al. 2006), our in vitro study has confirmed the regulation of GINS expression in response to the changes in oxygen or serum concentration. It is also noteworthy that GINS components were found to be more dynamically regulated by the microenvironment around cells, in contrast to the relatively constitutive expression of MCM2, another component of CMG complex. This dynamic regulation of GINS components would enable the tissue microenvironment to determine the proliferative state of tumor cells. Therefore, the inhibition of GINS induction can be a promising therapeutic strategy to control the growth and recurrence of glioblastoma. In addition, since GINS is an evolutionally conserved regulator of DNA replication which must be the common downstream of various signals regulating the growth of tumor cells with a variety of genetic abnormalities, GINS complex is expected to be the therapeutic target with a wide range of application, not only for glioblastoma but also for other intractable neoplasms.
